# Addition of Medicinal Plants Increases Antioxidant Activity, Color, and Anthocyanin Stability of Black Chokeberry (*Aronia melanocarpa*) Functional Beverages

**DOI:** 10.3390/plants11030243

**Published:** 2022-01-18

**Authors:** Desislava Teneva, Daniela Pencheva, Ani Petrova, Manol Ognyanov, Yordan Georgiev, Petko Denev

**Affiliations:** Laboratory of Biologically Active Substances, Institute of Organic Chemistry with Centre of Phytochemistry, Bulgarian Academy of Science, 139 Ruski Blvd., 4000 Plovdiv, Bulgaria; Desislava.Teneva@orgchm.bas.bg (D.T.); Daniela.Klisurova@orgchm.bas.bg (D.P.); Ani.Petrova@orgchm.bas.bg (A.P.); Manol.Ognyanov@orgchm.bas.bg (M.O.); Yordan.Georgiev@orgchm.bas.bg (Y.G.)

**Keywords:** black chokeberry (*Aronia melanocarpa*), anthocyanin stability, antioxidant activity, herbs, co-pigmentation, color stability, functional foods/beverages

## Abstract

The present study investigates the effect of the addition of medicinal plants, such as lady’s mantle, lavender, rosehip, and meadowsweet, on the chemical composition, antioxidant activity, and color intensity of ready-to-drink aronia nectar during pasteurization and long-term storage. Pasteurization caused a significant decrease in anthocyanin content of aronia nectar, which reduced to 20% of the initial value after four months of storage. Herbs provided different protection to aronia anthocyanins that degraded more slowly during the four-month storage compared to pasteurized control without herbs. The addition of medicinal plants enriched aronia nectar with phenolic compounds and increased its antioxidant activity by up to 52% in meadowsweet-aronia nectar. Moreover, it was accompanied by a color intensity magnification due to co-pigmentation of aronia anthocyanins and herbal phenolics. In contrast to anthocyanins, which constantly degraded during the whole period, color intensity began to stabilize after 30 days, demonstrating that co-pigmentation was progressively established during the time and rosehip provided the best stabilization of aronia nectar color. Current research demonstrates for the first time that medicinal plants such as lady’s mantle, rosehip, and especially meadowsweet can be used to increase antioxidant activity, color, and anthocyanin stability of black chokeberry functional beverages.

## 1. Introduction

*Aronia melanocarpa*, also known as black chokeberry, is a deciduous shrub that belongs to the *Rosaceae* family. Nowadays, chokeberries are cultivated as an important industrial crop and are processed into juices, nectars, wines, jams, and food-grade colorants. *Aronia melanocarpa* fruits are a good source of dietary fiber, vitamin B complex, carotenoids, tocopherols, vitamin C, and vitamin K, macroelements (K, Ca, P, Mg, Na), and microelements (Zn, Fe, Se, Cu, Mo, Cr, Mn, Si, Ni, B, V) [1]. Black chokeberry fruits reveal numerous health benefits and are among the richest sources of polyphenols and particularly anthocyanins in the plant kingdom [2,3]. Besides strong antioxidant and immunomodulatory activities, the beneficial health effects of chokeberry include gastroprotective, hypotensive, lipid-lowering, anticarcinogenic, neuroprotective, and cardioprotective effects [4,5,6,7,8]. Therefore, *Aronia melanocarpa* is recognized as a valuable medicinal plant [9]. The four major anthocyanins in *Aronia melanocarpa* are cyanidin-3-*O*-galactoside, cyanidin-3-*O*-arabinoside, cyanidin-3-*O*-glucoside, and cyanidin-3-*O*-xyloside [10]. In the past 20 years, the health benefits of anthocyanins have become a subject of numerous studies [11,12,13]. Anthocyanins act as antioxidants and show numerous health benefits, including anticancer, antiatherogenic, and anti-inflammatory effects [14]. Besides their health benefits, anthocyanins in foods are an important quality parameter that affects customers’ preferences. Anthocyanin stability in foods is affected by numerous factors, such as pH, solvents, temperature, and the presence of oxygen, enzymes, and other concomitant substances. The duration of heating during food processing has a strong influence on anthocyanin stability. Sadilova, Stintzing, and Carle have observed that elderberry anthocyanin content was very sensitive to thermal treatment and several studies reported anthocyanin reduction with an increase in temperature [15,16,17]. The heat-labile factors can force anthocyanin destruction, which could be accelerated by endogenous enzymes in fruits that cause pigment destruction during juice processing [17]. García-Viguera and Zafrilla have reported that temperature plays a critical role in anthocyanin loss during storage [18], whereas Kasparaviciene and Briedis revealed that eight-hour storage of black currant and black chokeberry juice concentrates at 60 °C resulted in a reduction in anthocyanins by 31% and 35%, and antioxidant activity decreased by 26% and 56%, respectively [19].

The combination of the attractive color of anthocyanins and their biological activities make the preservation of these pigments in foodstuffs and beverages an important technological problem. Co-pigmentation is one of the main mechanisms for the natural stabilization of anthocyanins. The co-pigmentation phenomenon occurs when a pigment (anthocyanin) and a co-pigment (colorless substance) form non-covalent complexes. It is observed as a bathochromic shift and hyperchromic effect, which stabilizes and modulates anthocyanin color, contributing to more intense color. However, it should be noted that most of the studies investigating co-pigmentation are performed in model systems, either with isolated and purified anthocyanins, or with model foods/beverages involving pure phenolic compounds [20,21].

As one of the richest sources of anthocyanins in the plant kingdom, black chokeberries and their products are thoroughly studied in regard to chemical composition and health benefits. However, studies regarding the stabilization of aronia anthocyanins through co-pigmentation are scarce. In our recent study, we investigated in model systems the co-pigmentation of anthocyanins isolated from black chokeberry with polyphenolic co-pigments and herbal extracts [22]. The investigated compounds provoked different co-pigmentation effects, accompanied by a color intensity (CI) magnification. The effect was profound at high co-pigment/pigment ratios, not characteristic for plant matter, and unachievable from a practical point of view. The addition of herbal extracts to purified anthocyanins led to a significant hyperchromic effect at much lower pigment/co-pigment ratios compared to pure compounds. Another study, using model beverages, revealed that chlorogenic acid enhanced the CI of aronia juice and that purified pigment from aronia had smaller co-pigmentation effects in comparison to fruit juice, indicating the participation of natural co-pigments present in fruits [23]. Furthermore, two studies investigated the effect of the addition of herbal extracts on the polyphenol content and composition of aronia juices without evaluating the effect on CI [24,25].

As a result of our previous study and literature review, we hypothesized that, except in model systems with purified anthocyanins, herbal extracts could exert their protective effect on black chokeberry anthocyanins and color stability in real food matrices. Moreover, this could be achieved in low concentrations, which are achievable in food processing, thus opening perspectives for potential practical application in the production of aronia-based functional foods. Therefore, the current study aimed to investigate the effect of the addition of herbs (lady’s mantle, lavender, rosehip, and meadowsweet) on the chemical composition, color stability, and antioxidant activity of ready-to-drink nectar from black chokeberry fruits after pasteurization and four-month storage. Namely, meadowsweet, lavender, and lady’s mantle extracts showed co-pigmentation with chokeberry anthocyanins in the above-mentioned study, whereas rosehip was chosen because of its high antioxidant potential and the observed synergistic effect in in vitro antioxidant activity, when mixed with aronia extracts [26]. Furthermore, it is known that co-pigmentation is progressively established during food storage and studies evaluating the co-pigmentation of aronia anthocyanins with herbal extracts during processing and storage of real food matrices such as aronia drinks are required [27].

## 2. Results and Discussion

### 2.1. Effect of Herb Addition on Chemical Composition, Color Intensity, and Antioxidant Activity of Black Chokeberry Nectar

In the European Union, there are strict regulations towards the requirements for fruit-based drinks, including fruit juices and fruit nectars [28]. According to them, fruit nectar is “The fermentable but unfermented product which is obtained by adding water with or without the addition of sugars and/or honey to fruit juice, to fruit purée and/or to concentrated fruit purée, and/or to a mixture of those products”. In our study, we chose four popular herbs to enrich aronia nectar with polyphenolic compounds and to investigate their effect on anthocyanin degradation and color stability after thermal pasteurization and four-month storage. The content of anthocyanins, polyphenols, as well as antioxidant activity and CI of black chokeberry nectar with 40% fruit content and aronia-herbal nectars are presented in Table 1. As it is evident from the results, black chokeberry nectar is a very rich source of polyphenols—3020.0 mg/L and particularly anthocyanins—700.4 mg/L, rendering very high oxygen radical absorbance capacity (ORAC) antioxidant activity of 63,878 µmol trolox equivalents (TE)/l. The addition of all herbs in a concentration of 2% significantly increased (*p* < 0.05) the polyphenol content of chokeberry nectar reaching 40%, when rosehip or meadowsweet extracts were added to the nectar. The increase in peroxyl radical scavenging activity (ORAC value) was even more significant (*p* < 0.05), exceeding 50% in the case of meadowsweet extract. Hydroxyl radical averting capacity (HORAC value) of chokeberry with meadowsweet was 29.6% higher than the pure chokeberry nectar (*p* < 0.05). Herb addition led to a decrease in anthocyanin content due to the soaking effect of aronia extract on dry herbs. As a result, CI of mixed aronia-herbal products decreased in all cases (*p* < 0.05), from 42.8 for pure aronia nectar to 36.8 for chokeberry with lavender nectar. The addition of herbs to aronia nectar was related to an increase in colored hue, which was significant (*p* < 0.05) for rosehip and lady’s mantle and could be attributed to carotenoid content of rosehip and brownish pigments in dry lady’s mantle aerial parts.

The addition of herbs to increase polyphenol content and antioxidant activity of aronia-based products have been investigated by other authors, as well. For example, Skapska et al. used *Cistus*, green tea, and nettle to fortify 80% aronia extracts with additional antioxidants [24]. Similar to our findings, the authors found that the addition of herbal extracts to aronia extract increases its antioxidant activity, measured by several in vitro assays, such as ORAC, ABTS, and DPPH. Moreover, a synergistic effect of selected herbal extracts was noted in total antioxidant capacity. In our study, the ORAC value of 40% aronia nectar was similar to their results for 80% aronia extract. This could be due to either difference in the raw material or the technological processing since our recent study demonstrated that black chokeberry fruits and functional drinks differ significantly in their chemical composition and antioxidant activity [29]. In another study, Sidor et al. investigated the effect of adding aqueous cinnamon and clove extracts on polyphenol loss in cloudy and clarified chokeberry juices [25]. The authors demonstrated that the addition of plant extracts prior to the pasteurization process influenced the content of phenolic compounds in the chokeberry juice and also observed a slight decrease in the anthocyanin content after the addition of herbal extracts.

In general, medicinal plants lack or are poor in anthocyanins, but are rich in other classes of phenolic compounds that could act as co-pigments to anthocyanins. The content of the major phenolic compounds of black chokeberry nectar, without and with herb addition, are presented in Table 2. Besides being a rich source of anthocyanins (cumulative content—700.4 mg/L), pure chokeberry nectar is a very rich source of several other classes of phenolic compounds (hydroxycinnamic acids, flavonols, and flavan-3-ols). Similarly to anthocyanin content, the content of other phenolic components such as neochlorogenic acid and quercetin decreased after the addition of herbs, again related to the soaking effect of aronia extract on dried herbs. On the other hand, the addition of herbs during extraction enriched chokeberry nectar with additional phenolics, such as *p*-coumaric and ellagic acids, and rosmarinic acid in the case of lavender (corresponding chromatograms are shown in Supplementary Figure S1). The addition of meadowsweet to aronia nectar increased significantly both the content of epicatechin from 42.4 mg/L to 115.3 mg/L (*p* < 0.05) and quercetin-3-glucoside from 116 mg/L to 145.5 mg/L, (*p* < 0.05), whereas the addition of 2% lady’s mantle during black chokeberry extraction increased the content of rutin in the product with 225% (*p* < 0.05). It is known that these flavonoids and particularly quercetin derivatives are strong chain-breaking antioxidants. Moreover, a recent study revealed that quercetin and epicatechin are the strongest antioxidants in black chokeberry fruits. However, due to the relatively low content, their contribution to the antioxidant activity of the fruits is not so significant compared to proanthocyanidins [3]. As it is demonstrated here, the addition of meadowsweet and lady’s mantle to aronia nectar enriched it in these flavonoids, which could explain the significantly higher ORAC activity of these two products when compared to pure aronia nectar (*p* < 0.05) (Table 1).

### 2.2. Changes in Chemical Composition, Color Intensity, and Antioxidant Activity of Black Chokeberry Nectar, without and with Herbs, during Pasteurization and Storage

#### 2.2.1. Changes in Anthocyanin Content and Polyphenol Constituents of Black Chokeberry Nectar, without and with Herbs, during Pasteurization and Storage

**Figure 1:**
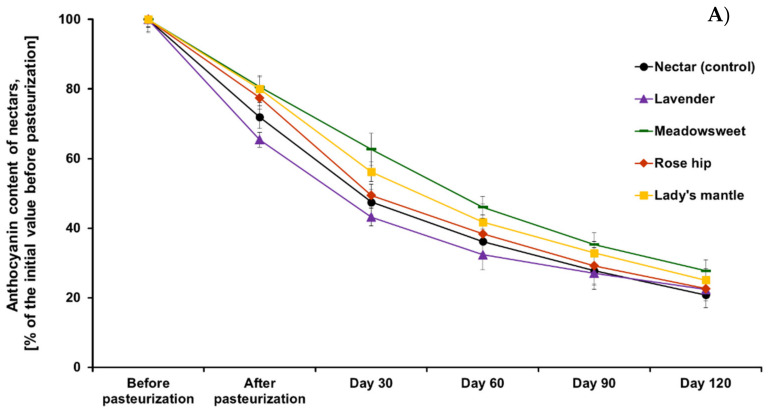

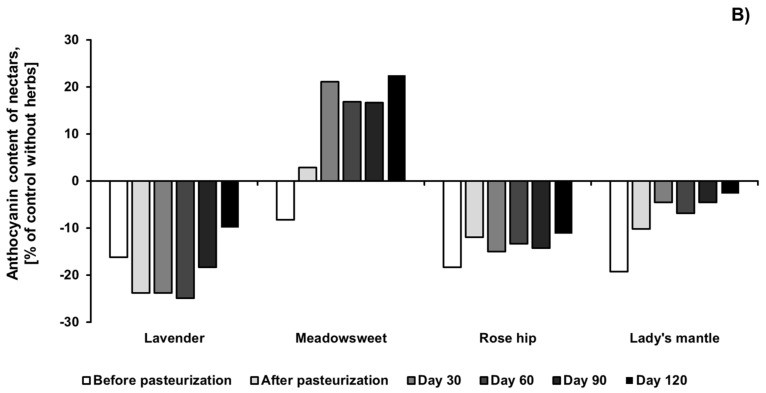
Changes of anthocyanin content of black chokeberry nectar, with or without the addition of herbs, after pasteurization and during storage: (**A**) expressed as a percent of the sample before pasteurization; (**B**) expressed as percent from the control (nectar without herb) at the corresponding time point.

Anthocyanins are particularly desired components in foods because on the one hand, they render the food an attractive red color, but on the other, due to their numerous health benefits, they increase functional and health-promoting properties of foods [22]. Because of their unstable nature, anthocyanins are readily destroyed during processing and storage, and the natural stabilization of anthocyanins with co-pigments is an important problem of practical significance. The rate of degradation of anthocyanins increases during processing and storage as temperature increases and follows first-order kinetics [30]. The increase in temperature causes hydrolysis of the glycosidic bonds, which leads to a loss in anthocyanin color and formation of brown chalcones [31]. Several studies have shown that aronia anthocyanins are prone to degradation during technological processing and especially heating. For example, Hwan and Ki investigated the effects of different factors (pH, temperature, light, sugars, organic acids, etc.) on the stability of anthocyanins extracted from *Aronia melanocarpa* and revealed that high temperatures and prolonged heating substantially reduced anthocyanin content [32]. Another study demonstrated that a decrease of anthocyanins in aronia drinks as a result of thermal pasteurization treatment could be diminished by high-pressure carbon dioxide preservation, which can be explained by a much lower heat dose during the latter process during storage [24]. Similar results were observed by Wilkes et al. after pasteurization of chokeberry juice. Moreover, anthocyanin losses were paralleled by increased polymeric color values, indicating that the small amounts of anthocyanins remaining were present in large part in polymeric forms [33]. Therefore, adding herbal extracts to anthocyanin-rich foods is an interesting approach to delay their degradation [34,35]. Figure 1 depicts the changes in anthocyanin content of the studied beverages after pasteurization (90°C, 10 min). Since co-extraction of chokeberry mesh and herbs led to a decrease of chokeberry nectar anthocyanin content, results are presented as percent of the initial anthocyanin content of the corresponding sample before pasteurization (Panel A) and compared to the content of anthocyanins in the control nectar without herb addition (Panel B). Our results showed that indeed anthocyanins in aronia nectar were degraded rapidly after thermal treatment at 90 °C and their degradation was constant during the whole monitoring period of 120 days, reaching about 20% of the initial value.

The addition of meadowsweet, rosehip, and lady’s mantle reduced anthocyanin degradation in all time points. Interestingly, the protective effect of the herbal extracts was most pronounced at shorter storage periods (30 days and 60 days), whereas after 120 days of storage differences in anthocyanin degradation were smaller. For example, anthocyanins from chokeberry nectar degraded with 32% and 19.8% less when meadowsweet was added in the extraction, respectively, after 30 and 60 days of storage. As it could be seen from Figure 1, Panel B, all herbs tended to protect black chokeberry anthocyanins during pasteurization and storage. However, the effect was most pronounced with meadowsweet. Although the initial content of anthocyanins in chokeberry-meadowsweet beverage was lower than that in pure aronia nectar, the content of anthocyanins immediately after pasteurization was higher than the control and increased significantly during storage. This is evidence that meadowsweet extract protected aronia anthocyanins from degradation during pasteurization and storage.

#### 2.2.2. Changes in Color Intensity of Black Chokeberry Nectar, without and with Herbs, during Pasteurization and Storage

Quite often, food color is only related to anthocyanin content, and CI or color expression is not taken into consideration. As already discussed, the co-pigmentation phenomenon could significantly increase CI at the same levels of anthocyanins in the sample [3]. However, it should be noted that most of the studies investigating co-pigmentation are in model systems with isolated and purified anthocyanins since real food matrices are more difficult to investigate, especially when herbs are added to the system. Usually, co-pigmentation is observed as a bathochromic shift and/or hyperchromic effect, which stabilizes and modulates anthocyanin color, contributing to a more intense color [20,21]. However, the addition of colored herbs during extraction could mask both hyperchromic and bathochromic effects. Therefore, we used CI as a marker for color expression. This method was proposed by Glories for the evaluation of wine color and later adopted in many studies [36,37,38,39].

**Figure 2:**
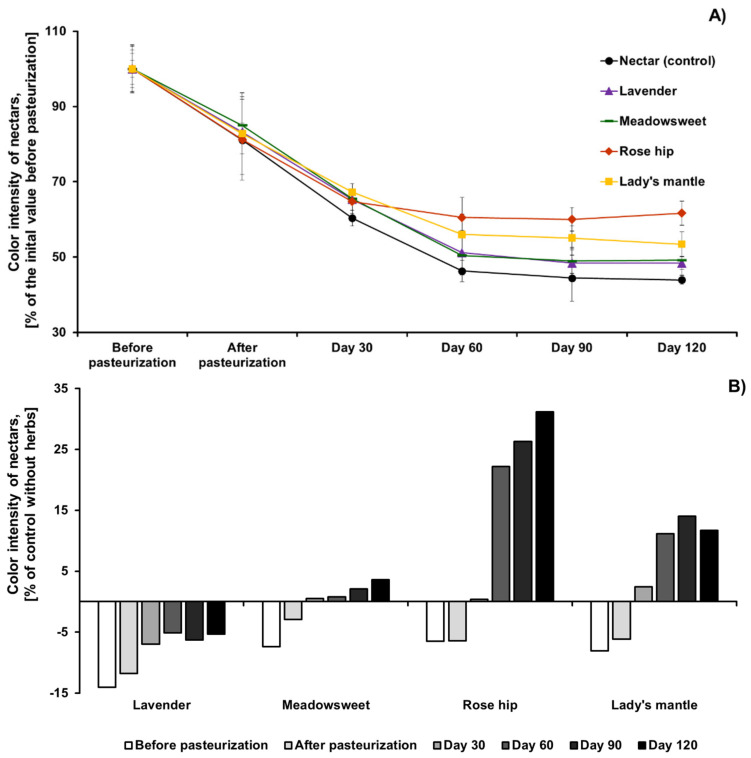
Changes in color intensity of black chokeberry nectar, with or without the addition of herbs, after pasteurization and during storage: (**A**) Expressed as percent before pasteurization; (**B**) Expressed as percent from the control (nectar without herb) at the corresponding time point.

The changes of CI of the studied beverages after thermal treatment and during storage are shown in Figure 2. Although anthocyanins are the main color pigments in the studied beverages, the trend of CI change was completely different from that of anthocyanin degradation. Thermal treatment led to a significant drop in CI (Figure 2, Panel A), probably due to anthocyanin degradation that occurred during pasteurization. In all cases, CI decreased within the first 30 days of storage. However, this process occurred at a slower rate in comparison to anthocyanin degradation (Figure 1). In the case of lavender, meadowsweet, and lady’s mantle, this decrease continued to the 60th day of storage, and after that, CI showed a trend towards stabilization. In the case of rosehip, stabilization of CI began after 30 days of storage. Due to the decreased anthocyanin content of mixed aronia-herbal beverages, their CI before pasteurization was lower than that of the nectar without herbs (Figure 2, Panel B). In all studied beverages, there was a trend for increased CI in comparison to control nectar. Although aronia-lavender nectar showed a trend for CI stabilization during storage, the absolute value of CI did not pass the value of the nectar at the corresponding time point. However, in all other cases (meadowsweet, rosehip, and lady’s mantle), after approximately 30 days of storage, the absolute values of CI exceeded that of the control nectar. For example, after 120 days of storage, aronia nectar with added rosehip had a CI that was 31.2% higher than that of the pure chokeberry nectar. Interestingly, the color of pure aronia nectar, which had the highest rate of anthocyanin degradation, also showed a trend for stabilization after approximately 60 days of storage. This is probably due to the high native content of hydroxycinnamic acids, epicatechin, and proanthocyanidins in aronia berries, which are strong co-pigments for aronia anthocyanins and could explain the elevated anthocyanin and color stability of aronia in comparison to other berries with non-acylated anthocyanins [22].

#### 2.2.3. Changes in Antioxidant Activity of Black Chokeberry Nectar, without and with Herbs, during Pasteurization and Storage

Measuring the antioxidant activity of foodstuffs is important from two aspects. Firstly, antioxidant components could protect food primary and secondary metabolites from oxidation, rancidity, and resulting degradation, and, therefore, preserve food quality. Secondly, food antioxidants taken with the diet could exert their antioxidant-related effects in the body, thus promoting human health [40]. Interestingly, we demonstrated that functional beverages obtained from the same batch of aronia fruits, which differed in their polyphenol content and antioxidant activity due to different technological processing, revealed different biological activity as well. This was demonstrated in a rat model of indomethacin-induced gastric ulcers, and the gastroprotective effect of aronia juices was related to their polyphenol content and antioxidant activity [5]. Furthermore, the effect was increased by enriching aronia juice with polyphenols from lady’s mantle.

**Figure 3:**
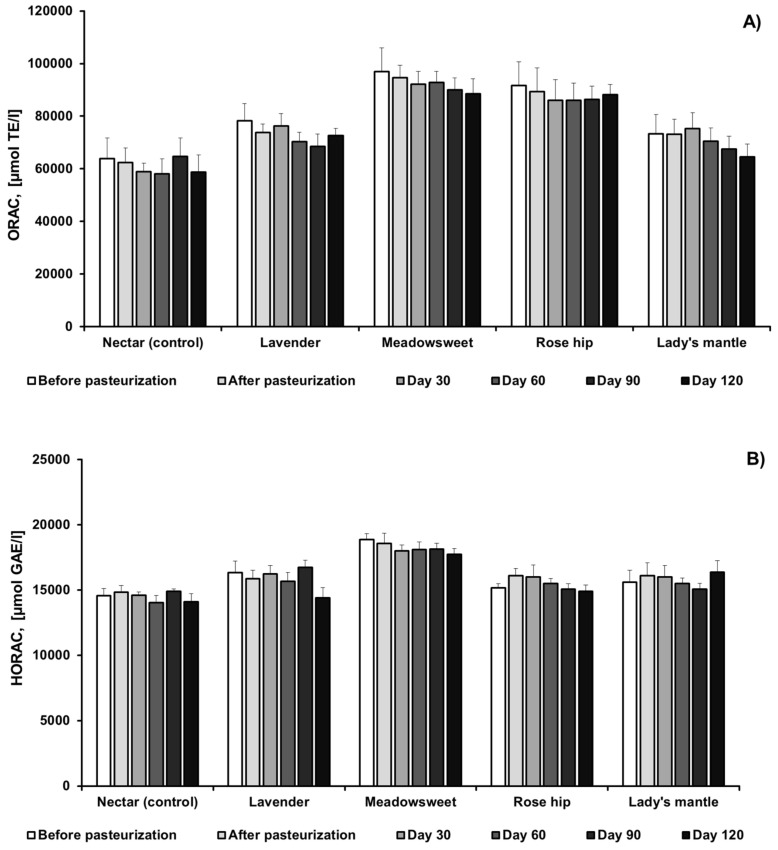

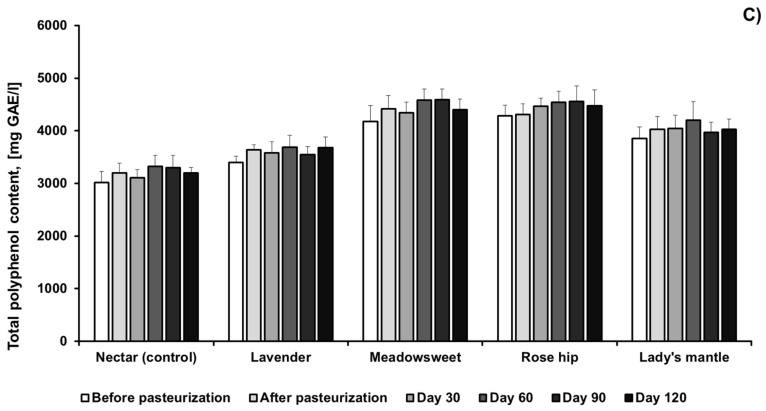
Changes in (**A**) ORAC (Oxygen radical absorbance capacity); (**B**) HORAC (Hydroxyl radical averting capacity) antioxidant activities and (**C**) total polyphenol content during thermal processing and storage of black chokeberry nectar with or without the addition of herbs. TE (trolox equivalents); GAE (Gallic acid equivalents). Results are presented as mean values ± SD.

In order to monitor the antioxidant activity changes after thermal treatment and during storage, we employed three different assays. ORAC is an indicator for the peroxyl radical scavenging capacity of antioxidants via hydrogen atom transfer, whereas analysis for total polyphenols relies on a single electron transfer. HORAC is an indicator of the ability of an antioxidant to prevent the formation of hydroxyl radicals during Fenton-like reactions [41]. Figure 3 presents data on the changes in antioxidant activity after thermal treatment and during 120 days of storage. As already stated, the addition of herbs significantly increased the polyphenol content of chokeberry nectar, and antioxidant activity measured by all three assays stayed comparatively stable during the whole period of storage and monitoring. These results correlate well with the total polyphenol content, which is kept more stable than anthocyanins during storage. From these results, it could be concluded that anthocyanin degradation did not significantly affect the antioxidant activity of aronia beverages. Anthocyanin degradation, caused by high temperature, results in the formation of benzoic acid derivatives and coumarins, with preserved hydroxyl groups and antioxidant activity [42,43]. In a previous study, we demonstrated that chokeberry proanthocyanidins were the major contributor to aronia antioxidant activity and, therefore, anthocyanin degradation did not significantly affect fruit antioxidant properties [3].

It is very difficult to relate the observed herb-induced magnification of anthocyanin or color stability with the observed antioxidant properties of aronia-herbal beverages. The extract of meadowsweet protected to the highest extent aronia anthocyanins from degradation during storage, but rosehip extract provided the best stabilization of aronia nectar color. Usually, there is a good correlation between polyphenol content and antioxidant activity, and it is known that certain phenolics could prevent or delay anthocyanin degradation [44]. Furthermore, the effectiveness of phenolic compounds as co-pigments is not related to the polyphenol content and resulting antioxidant properties, indicating that the qualitative phenolic composition of the extracts is more important than the total polyphenol content [22].

## 3. Materials and Methods

### 3.1. Chemicals

Cyanidin-3-*O*-galactoside chloride (≥97%), cyanidin-3-*O*-arabinoside chloride (≥97%), and cyanidin-3-*O*-glucoside chloride (≥97%) were purchased from Extrasynthese S.A. (Genay Cedex, France). Chlorogenic acid (≥95%), caffeic acid (≥98%), ferulic acid (≥99%), *p*-coumaric acid (≥98%), rutin (≥94%), epicatechin (≥90%), quercetin-3-*O*-glucoside (≥90%), quercetin (≥98%), rosmarinic acid (≥98%), ellagic acid (≥95%), neochlorogenic acid (≥98%), gallic acid (100%), Trolox (≥98%), fluorescein (FL) disodium salt (100%), and 2,2′-Azobis(2-amidinopropane) dihydrochloride (AAPH) (≥98%) were purchased from Sigma-Aldrich (Steinheim, Germany) and Folin–Ciocalteu’s phenol reagent was purchased from Merck (Darmstadt, Germany). All other solvents used were of analytical grade and purchased from local distributors.

### 3.2. Plant Materials

Black chokeberry fruits were cultivated from Dimitar Sokolov (Gotse Delchev, Latitude: 41.5667, Longitude: 23.7333, Bulgaria) and harvested in the stage of full maturity in August 2020. Fresh fruits were frozen and stored at −18 °C by Vitanea Ltd. (Plovdiv, Bulgaria) and delivered frozen prior to extraction and analysis.

Cut herbs (size 1–4 mm): common lavender (*Lavandula angustifolia* Mill.) aerial parts, lady’s mantle (*Alchemilla glabra* Neygenf.) aerial parts, and meadowsweet (*Filipendula ulmaria* L.) aerial parts, produced by Herbal pharmacy No. 1, Plovdiv were purchased from “Pharmacy 36.6”, Plovdiv, Bulgaria. Dried deseeded rosehips (*Rosa canina* L.) fruits (Size 2–8 mm, batch number RSPLH01032021L) were purchased from Balevski and Kirov Ltd. (Tryavna, Gabrovo region, Bulgaria).

### 3.3. Preparation of Beverages

Black chokeberry nectar with 40% fruit content was prepared according to a previously reported procedure [29]. Briefly, frozen aronia fruits were defrosted at room temperature and homogenized in a laboratory blender. After that, 400 g of fruit homogenate were mixed with 600 mL ultrapure water, transferred to a brown-glass bottle, and incubated in a thermostatic water bath shaker (NUVE, Asagi Ovecler Ankara, Turkey) for 1 h, at 60 °C. After that, solid fruit residue was separated from the clear aronia nectar by pressing through a cheesecloth.

For the preparation of mixed aronia-herbal beverages, 400 g of fruit homogenate were mixed with 600 mL ultrapure water and 20 g (2% *w*/*v*) of the respective herb (lavender, lady’s mantle, meadowsweet, or rosehip). Mixtures were transferred in brown-glass bottles and incubated in a thermostatic water bath shaker (NUVE, Asagi Ovecler Ankara, Turkey) for 1 h, at 60 °C. After that, solid fruit-herbal residues were separated from the clear nectars by pressing through a cheesecloth.

All extraction procedures were repeated 5 times in order to collect approximately 3.5 l of all nectars necessary for the whole experiment. 50 mL from all samples were separated for analysis before thermal treatment, whereas the remaining amounts were subjected to pasteurization.

### 3.4. Pasteurization and Storage of Black Chokeberry Nectars with or without Herb Addition

Pasteurization of fruit nectars was performed according to Rabie et al., with slight modifications [45]. Briefly, filtrated nectars were poured in 250 mL glass bottles, capped with twist-off metal caps, transferred into a 90 °C water bath, and pasteurized for 10 min minutes. Pasteurized nectars were cooled to room temperature in a cold water bath for 30 min. In total, 10 bottles were kept from each nectar—2 parallel samples for each time point (after pasteurization; and 30, 60, 90, and 120 days of storage).

Pasteurized bottles were kept in a dark place at 20.0 ± 1.0 °C for the whole storage period. Two bottles from each sample/nectar were randomly chosen, either after pasteurization or at the corresponding time points of storage, and opened immediately before analysis.

### 3.5. Color Evaluation

CI and color hue were calculated according to Bimpilas et al. via the following formulas [37]:

CI = A_420_ + A_520_ + A_620_

Color hue = A_420_/A_520_

where: A_420_, A_520,_ and A_620_ are the absorbance values measured at 420 nm, 520 nm, and 620 nm, respectively.

Using this method, the absorbance of samples was measured at 420 nm (yellow), 520 nm (red), and 620 nm (blue), thus reflecting the% contribution of different pigment categories to the expressed color. All spectrophotometric measurements were performed at a Biowave DNA spectrophotometer using a 0.05 mm optical path glass cell (Biochrom WPA, Cambridge, United Kingdom). Results for color intensity and color hue were recalculated for 1 mm optical path.

### 3.6. Total Polyphenol Content Analysis

Total polyphenols were determined according to the method of Singleton and Rossi, with the Folin–Ciocalteu’s reagent [46]. Gallic acid was employed as a calibration standard, and results were expressed in mg gallic acid equivalents (GAE) per liter of extract ± SD, (n = 6).

### 3.7. HPLC Determination of Anthocyanins

The quantitation of anthocyanins was conducted on a Nexera-i LC2040C Plus UHPLC system (Shimadzu Corporation, Kyoto, Japan) with a UV detector and a binary pump. The system was controlled by LabSolutions (ver. 5.98) software (Shimadzu Corp.). A wavelength of 520 nm was used. Anthocyanins were separated using an Agilent TC-C18 column (5 μm, 4.6 × 250 mm) at 25 °C. The following mobile phases were used: 5% formic acid (A) and 100% methanol (B) at a flow rate of 1.0 mL/min. The gradient condition started with 15% B and linearly increased to 30% B at 20 min. The sample injection volume was 20 μL. The results were calculated from the relationship between the peak area response and concentration, using linear regression for each analyte. The R-squared values (R^2^) were >0.99 for all calibration curves. Anthocyanins were identified by comparing the retention times of unknown analytes with analytical grade standards (cyanidin-3-*O*-galactoside, cyanidin-3-*O*-arabinoside, and cyanidin-3-*O*-glucoside). The total anthocyanin content was expressed as the sum of the content of the three anthocyanins and expressed as mg per liter of extract ± SD (n = 4).

### 3.8. HPLC Analysis of Phenolic Compounds

The main polyphenol compounds of aronia and herbal extracts were quantified on a Nexera-i LC2040C Plus UHPLC system (Shimadzu Corporation, Kyoto, Japan) with a UV detector and a binary pump. A wavelength of 280 nm was used. The separation of phenolics was performed on an Agilent TC-C18 column (5 μm, 4.6 mm × 250 mm) at 25 °C. The mobile phases constituted 0.5% acetic acid (A) and 100% acetonitrile (B) at a flow rate of 0.8 mL/min. The gradient condition started with 14% B, between 6 min and 30 min linearly increased to 25% B, then to 50% B at 40 min. The sample injection volume was 20 μL. The results were calculated from the relationship between the peak area response and concentration, using linear regression for each analyte. The R-squared values (R^2^) were >0.99 for all calibration curves. All phenolic compounds were identified by comparing the retention times of unknown analytes with analytical grade standards (chlorogenic acid, caffeic acid, ferulic acid, *p*-coumaric acid, rutin, epicatechin, quercetin-3-*O*-glucoside, quercetin, rosmarinic acid, ellagic acid, neochlorogenic acid) and expressed as mg per liter of extract ± SD (n = 4).

### 3.9. Oxygen Radical Absorbance Capacity (ORAC) Assay

Oxygen Radical Absorbance Capacity was measured according to the method of Ou, Hampsch-Woodill, and Prior [47] with some modifications [48]. Solutions of AAPH, FL, and trolox were prepared in a phosphate buffer (75 mmol/L, pH 7.4). Samples were diluted in the phosphate buffer as well. The reaction mixture (total volume 200 μL) contained FL—(170 μL, final concentration 5.36 × 10^−8^ mol/L), AAPH—(20 μL, final concentration 51.51 mmol/L), and sample—10 μL. The FL solution and sample were incubated at 37 °C for 20 min directly in a microplate reader, and AAPH (dissolved in buffer at 37 °C) was added. The mixture was incubated for 30 s before the initial fluorescence was measured. After that, the fluorescence readings were taken at the end of every cycle (1 min) after shaking. For the blank, 10 μL of phosphate buffer was used instead of the extract. Trolox solutions (6.25; 12.5; 25 and 50 μmol/L) were used for defining the standard curve. ORAC was measured using a FLUOstar OPTIMA plate reader (BMG Labtech, Germany), excitation wavelength of 485 nm and emission wavelength of 520 nm were used. ORAC values were expressed in µmol TE per liter of extract ± SD (n = 8).

### 3.10. Hydroxyl Radical Averting Capacity (HORAC) Assay

HORAC was performed as described by Ou et al. [49]. Briefly, hydrogen peroxide solution of 0.55 M was prepared in distilled water. 4.6 mM Co(II) was prepared as follows: 15.7 mg of CoF_2_·4H_2_O and 20 mg of picolinic acid were dissolved in 20 mL of distilled water. FL—170 μL (60 nM, final concentration) and 10 µL of the sample were incubated at 37 °C for 10 min directly in the FLUOstar plate reader. After incubation 10 μL H_2_O_2_ (27.5 mM, final concentration) and 10 μL of Co(II) (230 µM final concentration) solutions were subsequently added. The initial fluorescence was measured, after which the readings were taken every minute after shaking. For the blank sample, a phosphate buffer solution was used. 100, 200, 600, 800, and 1000 μM gallic acid solutions (in phosphate buffer 75 mM, pH = 7.4) were used for building the standard curve. Measurements were performed on a FLUOstar OPTIMA fluorometer (BMG LABTECH, Offenburg, Germany). The excitation wavelength of 485 nm and emission wavelength of 520 nm was used. The results were expressed in micromole gallic acid equivalents (µmol GAE) per liter of extract ± SD (n = 8).

### 3.11. Statistical Analysis

All samples were prepared and analyzed in duplicates. The HPLC analyses were performed twice for every single sample (n = 4), whereas other analyses were run at least in triplicates for each sample (n = 6). Results were expressed as mean values ± standard deviations. One-way analysis of variance (ANOVA) and Student’s t-test were used to evaluate the differences of the mean between groups. *p* values less than 0.05 were considered to be significant. Microsoft Excel, 2013 (Microsoft Corporation, Redmond, WA, USA) was used in the analyses.

## 4. Conclusions

The current work provides evidence for the first time that the addition of herbs during the processing of black chokeberry fruits can successfully increase polyphenol content, antioxidant activity, color, and anthocyanin stability of resulting functional beverages, thus increasing their functionality and consumers’ acceptance. However, different herbs provided different protective effects. Meadowsweet extracts protected to the highest extent aronia anthocyanins from degradation, but rosehip provided the best stabilization of aronia nectar color during storage. Therefore, each herbal material should be investigated for its ability to increase anthocyanin and/or color stability of foods. Interestingly, CI decreased with a different pattern from anthocyanin degradation, thus revealing that the co-pigmentation effect in chokeberry nectar with herbal phenolics was established after the first month of beverage storage. Our research findings could find practical application in the industrial production of aronia-based functional drinks with enhanced antioxidant activity and stabilized anthocyanins, and color for the prevention and supplementation of oxidative stress-related diseases.

## Figures and Tables

**Table 1:** Anthocyanin and polyphenol content, antioxidant activity, color intensity, and color hue of black chokeberry nectar with or without herb addition.

	Chokeberry	Chokeberry with Lavender	Chokeberry with Meadowsweet	Chokeberry with Rosehip	Chokeberry with Lady’s Mantle
Anthocyanin content, mg/L	700.4 ^c^ ± 16.0	587.0 ^a^ ± 12.5	642.9 ^b^ ± 13.5	571.9 ^a^ ± 19.7	565.2 ^a^ ± 21.1
Total polyphenol content, mg/L	3020.0 ^a^ ± 65.4	3395.7 ^b^ ± 59.4	4177.2 ^d^ ± 120.3	4281.4 ^d^± 88.7	3851.5 ^c^ ± 53.3
ORAC ^1^, µmol TE/L	63,878 ^a^ ± 190	73,198 ^b^ ± 1750	96,973 ^d^ ± 1488	91,713 ^d^ ± 3322	78,227 ^c^ ± 2319
HORAC ^2^, µmol GAE ^3^/L	14,556 ^a^ ± 32	16,336 ^c^ ± 54	18,860 ^d^ ± 745	15,158 ^b^ ± 873	15,612 ^b^ ± 203
CI ^4^	42.8 ^b^ ± 1.0	36.8 ^a^ ± 2.3	39.6 ^a^ ± 2.4	40.0 ^a^ ± 1.6	39.3 ^a^ ± 1.6
Color hue	9.0 ^ab^ ± 0.8	9.8 ^abc^ ± 0.6	8.7 ^a^ ± 0.4	11.7 ^d^ ± 1.0	10.7 ^cd^ ± 0.6

^1^ Oxygen radical absorbance capacity; ^2^ Hydroxyl radical averting capacity; ^3^ Gallic acid equivalents; ^4^ Color intensity. Results are presented as mean values ± standard deviation (SD). There are no significant differences among values marked with the same superscript letters in individual lines.

**Table 2:** Major phenolic constituents (mg/L) of black chokeberry nectar with or without herb addition.

	Chokeberry	Chokeberry with Lavender	Chokeberry with Meadowsweet	Chokeberry with Rosehip	Chokeberry with Lady’s Mantle
Gallic acid	-	-	34.9 ^a^ ± 3.4	57.2 ^b^ ± 4.8	32.4 ^a^ ± 4.1
Neochlorogenic acid	322.1 ^b^ ± 18.5	242.1 ^a^ ± 12.4	273.3 ^a^ ± 16.5	254.3 ^a^ ± 21.0	240.2 ^a^ ± 20.3
Chlorogenic acid	269.1 ^b^ ± 11.2	181.6 ^a^ ± 10.8	280.6 ^b^ ± 19.6	273.8 ^b^ ± 28.1	283.1 ^b^ ± 21.2
Caffeic acid	-	19.9 ^a^ ± 2.6	-	-	-
Epicatechin	42.4 ^a^ ± 3.1	50.7 ^ab^ ± 4.8	115.3 ^c^ ± 9.6	53.9 ^b^ ± 4.1	53.7 ^b^ ± 2.3
*p*-Coumaric acid	-	17.4 ^b^ ± 1.2	16.9 ^b^ ± 2.1	11.7 ^a^ ± 0.9	20.5 ^b^ ± 0.8
Ferulic acid	-	22.1 ^a^ ± 0.8	58.3 ^c^ ± 3.1	45.2 ^b^ ± 2.1	47.2 ^b^ ± 4.0
Rutin	178.0 ^a^ ± 8.1	124.7 ^a^ ± 9.6	188.4 ^b^ ± 9.2	172.8 ^b^ ± 5.1	579.8 ^c^ ± 14.6
Ellagic acid	-	-	34.2 ^a^ ± 1.2	49.5 ^b^ ± 2.9	65.9 ^c^ ± 4.1
Quercetin-3-glucoside	116.0 ^a^ ± 7.2	132.7 ^ab^ ± 5.6	176.2 ^c^ ± 9.9	145.5 ^b^ ± 13.1	118.8 ^a^ ± 10.5
Rosmarinic acid	-	29.2 ^a^ ± 0.2	-	-	-
Quercetin	14.5 ^b^ ± 0.8	6.2 ^a^ ± 0.2	12.3 ^b^ ± 0.6	8.7 ^a^ ± 0.9	8.4 ^a^ ± 1.0

Results are presented as mean values ± SD. There are no significant differences among values marked with the same superscript letters in individual lines (*p* < 0.05).

## Data Availability

Data is contained within the article.

## Supplementary Materials

The following supporting information can be downloaded at: https://www.mdpi.com/article/10.3390/plants11030243/s1, Figure S1. UHPLC elution pattern of phenolic constituents: (A) chokeberry with meadowsweet; (B) chokeberry with lavender; (C) chokeberry with rose hip; (D) chokeberry with lady’s mantle; (E) chokeberry (control).
