# Supplementary material for: Addition of Medicinal Plants Increases Antioxidant Activity, Color, and Anthocyanin Stability of Black Chokeberry (Aronia melanocarpa) Functional Beverages
Source: Plants (Basel). 2022 Jan 18;11(3):243. doi: 10.3390/plants11030243 (PMC8838913; doi:10.3390/plants11030243)
Supplement: Supplementary file 1 [file plants-11-00243-s001.zip › plants-1544208-supplementary.pdf]

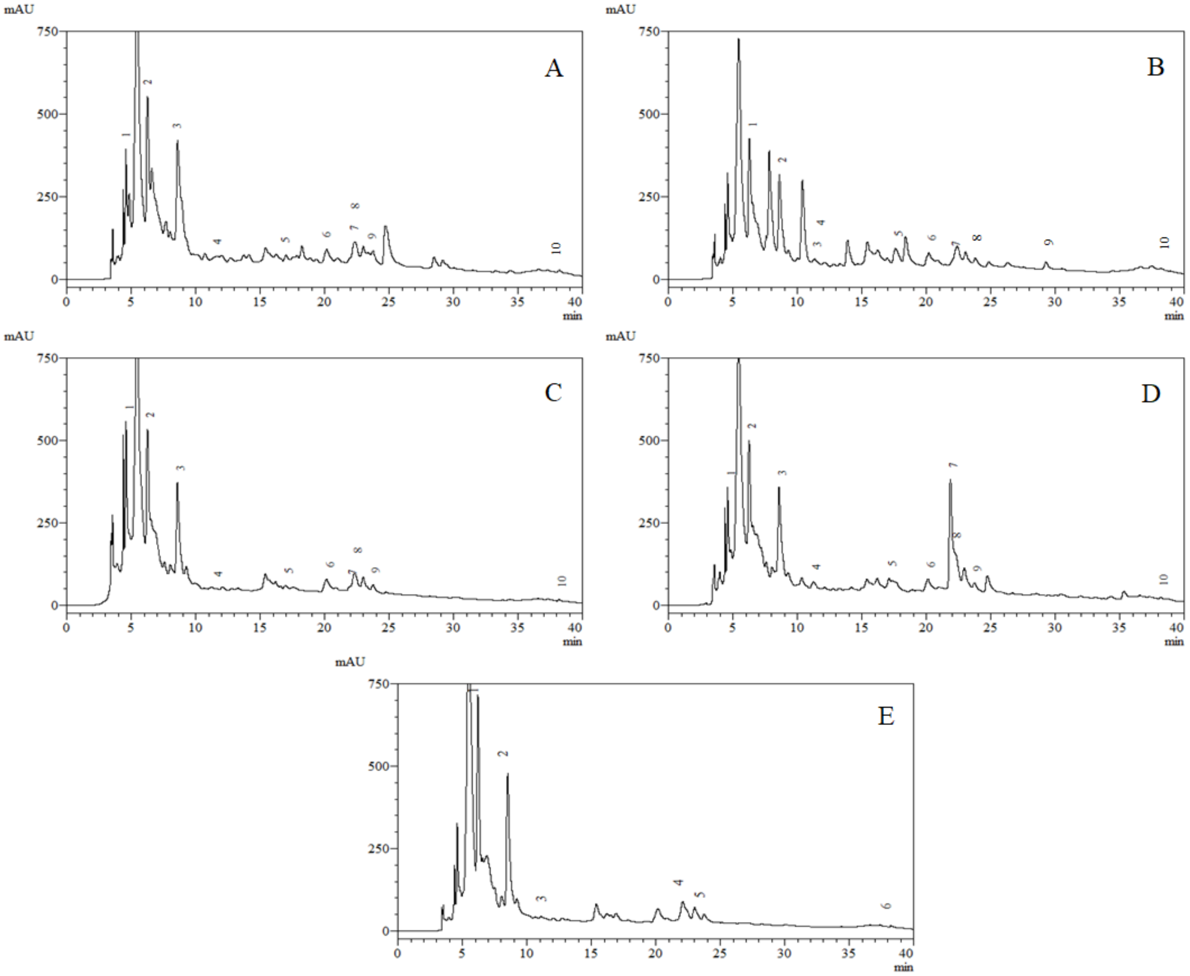

Figure S1. UHPLC elution pattern of phenolic constituents: A) chokeberry with meadowsweet; B) chokeberry with lavender; C) chokeberry with rose hip; D) chokeberry with lady's mantle; E) chokeberry (control).

A) 1 – gallic acid; 2 – neochlorogenic acid; 3 – chlorogenic acid; 4 – epicatechin; 5 – *p*-coumaric acid; 6 – ferulic acid; 7 – rutin; 8 – ellagic acid; 9 – quercetin-3-glucoside; 10 – quercetin

B) 1 – neochlorigenic acid; 2 – chlorogenic acid; 3 – caffeic acid; 4 – epicatechin; 5 – *p*-coumaric acid; 6 – ferulic acid; 7 – rutin; 8 – quercetin-3-glucoside; 9 – rosmaricnic acid; 10 – quercetin

C and D) 1 – gallic acid; 2 – neochlorogenic acid; 3 – chlorogenic acid; 4 – epicatechin; 5 – *p*-coumaric acid; 6 – ferulic acid; 7 – rutin; 8 – ellagic acid; 9 – quercetin-3-glucoside; 10 – quercetin

E) 1 – neochlorogenic acid; 2 – chlorogenic acid; 3 – epicatechin; 4 – rutin; 5 – quercetin-3-glucoside; 6 – quercetin
